# Systemic cytokine profiles in biliary atresia

**DOI:** 10.1371/journal.pone.0267363

**Published:** 2022-04-22

**Authors:** Wanvisa Udomsinprasert, Tachatra Ungsudechachai, Paisarn Vejchapipat, Yong Poovorawan, Sittisak Honsawek

**Affiliations:** 1 Department of Biochemistry, Faculty of Pharmacy, Mahidol University, Bangkok, Thailand; 2 Department of Surgery, Faculty of Medicine, King Chulalongkorn Memorial Hospital, Thai Red Cross Society, Chulalongkorn University, Bangkok, Thailand; 3 Center of Excellence in Clinical Virology, Department of Pediatrics, Faculty of Medicine, King Chulalongkorn Memorial Hospital, Chulalongkorn University, Bangkok, Thailand; 4 Department of Biochemistry, Osteoarthritis and Musculoskeleton Research Unit, Faculty of Medicine, King Chulalongkorn Memorial Hospital, Thai Red Cross Society, Chulalongkorn University, Bangkok, Thailand; Texas A&M University, UNITED STATES

## Abstract

**Background:**

Inflammation and immune dysregulation persuade biliary duct injury in biliary atresia (BA), a leading cause of pediatric liver transplantation given lack of specific biomarkers. We aimed to determine associations between systemic cytokine profiles and clinical parameters in BA patients and to identify potential BA biomarkers.

**Methods:**

Systemic levels of 27 cytokines were measured in 82 BA patients and 25 healthy controls using a multiplex immunoassay. Relative mRNA expressions of candidate cytokines in 20 BA livers and 5 non-BA livers were assessed using quantitative real-time PCR.

**Results:**

Higher levels of 17 cytokines including IL-1β, IL-6, IL-7, IL-8, IL-9, IL-2, IL-15, eotaxin, IP-10, MCP-1, MIP-1α, MIP-1β, G-CSF, IL-1ra, IL-4, IL-5, and IL-10 and lower levels of IFN-α and PDGF were significantly associated with BA. In BA patients, increased levels of IL-7, eotaxin, IP-10, and IL-13 were significantly associated with unfavorable outcomes including jaundice, fibrosis, and portal hypertension. Indeed, systemic levels of those cytokines were significantly correlated with clinical parameters indicating jaundice, fibrosis, and hepatic dysfunction in BA patients. Out of 27 cytokines, 4 (IL-8, IP-10, MCP-1, and PDGF) had potential as sensitive and specific biomarkers of BA. Of these, higher IL-8 levels were significantly associated with reduced survival of BA. In BA livers, relative mRNA expressions of *IL-8*, *IP-10*, and *MCP-1* were significantly up-regulated.

**Conclusions:**

Higher levels of several cytokines including inflammatory cytokines, immunomodulatory cytokines, chemokines, and anti-inflammatory cytokines and lower levels of growth factors would reflect inflammatory and immune responses related to BA development. Among 27 cytokines, plasma IL-8 might have great potential as a diagnostic and prognostic biomarker for BA.

## Introduction

Biliary atresia (BA) is a serious neonatal liver disease with sclerosing cholangiopathy of complex pathogenesis, which is characterized by a fibro-inflammatory obliteration of the extrahepatic bile ducts leading to severe cholestasis, progressive liver fibrosis, and eventually to end-stage liver failure [1]. Despite its rarity, BA is the most common reason for pediatric liver transplantation. Although Kasai portoenterostomy (KPE), regarded as the first-line operation, can restore bile drainage and is essential for survival, in most patients, it does not halt progressive liver fibrosis [2], a key determinant of transplant-free survival, because of delayed diagnosis and imperfect non-invasive indicators. In this regard, it is worth noting that a new, non-invasive diagnostic marker may expedite the differential diagnosis and better enable the assessment of postoperative prognosis, which may pave the way for improving clinical outcomes of BA patients following KPE or even avoiding the need for liver transplantation. Molecular identification of BA pathogenesis is therefore of paramount clinical importance for developing reliable biomarkers.

Of several pathological features involved in BA etiology, the innate and adaptive immune responses are considered to play an important role in progression of biliary tract injury predominating in BA [3]. Given that cytokines, soluble polypeptides secreted by a wide range of cells, function as a vital player in immunological and inflammatory responses in the systemic and local environments, alterations in plasma levels of those molecules have been suggested as potential biomarkers of tissue injury–especially liver injury [4–6]. As to their biological roles, pro-inflammatory cytokines including interleukin (IL)-1β, IL-6, and tumor necrosis factor (TNF)-α, produced predominantly by activated macrophages, can stimulate the recruitment of inflammatory cells. Through paracrine and autocrine pathways, they subsequently activate inflammatory cells to produce other cytokines known as chemokines that are directly chemotactic to leukocytes and stromal cells, leading to production of tissue-damaging mediators responsible for liver fibrosis as a wound-healing process [7, 8]. In post-operative BA patients, it has been demonstrated that progression of hepatic inflammation is characterized by excessive production of cytokines including pro-inflammatory cytokines, T-helper (Th) cytokines, and macrophage cytokines [9]. Over the past decades, an increasing number of studies have attempted to link the systemic and local levels of various cytokines including pro-inflammatory cytokines (IL-1β, IL-6, TNF-α), immunomodulatory cytokines consisting of Th-1 cytokines (IL-2, interferon (IFN)-γ) and Th-2 cytokines (IL-4, IL-10, IL-12p70, IL-12p40), chemokine (IL-8), and macrophage cytokines [IL-18, transforming growth factor (TGF)-β] to BA severity [9–13]. Altogether, the aforementioned results lend further support to the view that plasma cytokines may serve as non-invasive biomarkers for the disease progression in post-operative BA patients.

Although changes in plasma levels of cytokines in BA patients have been thoroughly explored, no attempt has been made to capture the breadth of profiles of 27 systemic cytokines in BA patients, in addition to relationships between systemic cytokine profiles and clinical parameters of BA patients–especially liver fibrosis. Accordingly, the objective of our study was to determine: (1) systemic cytokine profiles in BA patients and healthy controls; (2) whether systemic levels of cytokines were associated with clinical parameters of BA patients and can be a useful diagnostic tool to detect the disease progression; and (3) mRNA expressions of candidate cytokines derived from cytokine profiles in BA livers compared with non-BA livers.

## Materials and methods

This study protocol was approved by the Institutional Review Board of the Faculty of Medicine, Chulalongkorn University and the Faculty of Dentistry/Faculty of Pharmacy, Mahidol University and conducted in accordance with the ethical standards outlined in the Declaration of Helsinki. Written informed consent was obtained from all participants’ guardian.

### Study participants

A total of 107 study subjects (82 BA patients and 25 age-matched healthy controls) were enrolled in this case-control study. All BA patients were diagnosed by intraoperative cholangiography and were surgically treated with original Kasai operation. Healthy controls who attended the Well Baby Clinic at King Chulalongkorn Memorial Hospital for vaccination had normal physical findings and no underlying disease. According to serum levels of total bilirubin (TB) indicating severity of cholestasis, BA patients were stratified into non-jaundice (TB <2 mg/dL, *n* = 51) and persistent jaundice groups (TB ≥2 mg/dL, *n* = 31). In the context of severity of liver fibrosis (liver stiffness values), the patients were also categorized into no fibrosis (<7.1 kPa, *n* = 15) and significant fibrosis groups (≥7.1 kPa, *n* = 67). This cut-off value is based on previous studies that proposed the optimal cut-off value as 7.0 kPa to diagnose significant liver fibrosis (≥ F2) in general population [14] and patients with non-alcoholic fatty liver disease [15]. In terms of portal hypertension (PH) indicated by the presence of ascites and/or esophageal varices observed on endoscopy, BA patients were classified into non-PH (*n* = 37) and PH (*n* = 45).

### Assessment of systemic cytokine profiles

Venous blood was collected from healthy controls and BA patients at the time of KPE into a sterile ethylenediamine tetraacetic acid (EDTA)-containing tube. Plasma samples were separated by centrifugation at 1,500 g for 10 min and subsequently stored at −80°C for subsequent analysis. Systemic concentrations of cytokines in BA patients and healthy controls were measured using the Bio-Plex Pro Human Cytokine 27-Plex Assay on the Bio-Rad MAGPIX Multiplex Reader (Bio-Rad, Hercules, CA, USA) following the manufacturer’s instructions. The analyzed cytokines were as follows: (1) inflammatory cytokines including IL-1β, IL-6, IL-7, IL-8, IL-9, and TNF-α; (2) immunomodulatory cytokines including IL-2, IL-12p70, IL-15, IL-17, and IFN-γ; (3) chemokines including eotaxin, IFN-γ-induced protein 10 (IP-10), monocyte chemoattractant protein 1 (MCP-1), macrophage inflammatory protein (MIP)-1α, MIP-1β, and RANTES (Regulated on Activation, Normal T Expressed and Secreted, CCL5); (4) growth factors including granulocyte colony stimulating factor (G-CSF), granulocyte macrophage colony-stimulating factor (GM-CSF), basic fibroblast growth factor (bFGF), platelet-derived growth factor (PDGF), and vascular endothelial growth factor (VEGF); and (5) anti-inflammatory cytokines including IL-1 receptor antagonist (IL-1ra), IL-4, IL-5, IL-10, and IL-13.

### Determination on mRNA expression of candidate cytokines

As the cornerstone of the diagnostic work-up of infants with undiagnosed cholestasis, perioperative liver biopsy was undertaken during an operation to emphasize timely recognition of biliary obstruction and early KPE in BA patients. Available liver specimens from 20 out of 82 BA patients and 5 non-BA patients who suffered from choledochal cysts and underwent liver biopsy without signs of fibrosis were harvested at the Department of Surgery, King Chulalongkorn Memorial Hospital. Total RNA was extracted from the liver biopsies using a RNeasy Mini Kit (Qiagen, Hilden, Germany) with cDNA reverse transcribed using TaqMan Reverse Transcription Reagents (Applied Biosystems, Inc., Foster City, CA, USA). Real-time polymerase chain reaction (PCR) was performed using SYBR Green fluorescence (biotechrabbit GmbH, Hennigsdorf, Germany) on a StepOnePlus Real-Time PCR System (Applied Biosystems, Inc., Foster City, CA, USA). Relative mRNA expressions of candidate cytokines including *IL-8*, *IP-10*, *MCP-1*, and *PDGF* were normalized to glyceraldehyde-3-phosphate dehydrogenase (*GAPDH*) as an internal control and were determined using the 2^-ΔΔCt^ method.

### Statistical analysis

All statistical analyses were performed using SPSS Statistics version 26.0 (SPSS Inc., Chicago, IL, US) and GraphPad Prism 8.0 (GraphPad Software, Inc., CA, US). Differences in demographic and clinical characteristics between groups were estimated using chi-square (χ^2^) test for categorical variables represented as frequencies (percentages) and Mann Whitney *U* test for abnormally distributed continuous variables represented as median with interquartile ranges (IQR, Q1-Q3). Correlations between BA severity and plasma levels of cytokines were evaluated using Spearman’s rho correlation coefficient (*r*). To exclude or control confounding variables including age, gender, and body mass index (BMI) that can interfere the outcome, multivariate logistic regression models were performed. Kaplan-Meier analysis with end points of death was undertaken to estimate the survival function, in which the differences in survival curves were determined using Log-rank test. Receiver operating characteristic (ROC) curve was constructed for the estimation of the area under the ROC curve (AUC), sensitivity, and specificity, indicating the feasibility of using plasma levels of cytokines as possible biomarkers for BA. Data are presented as mean ± standard deviation (SD) and median with IQR (Q1-Q3). For all analyses, a *P*-value of less than 0.05 (based on a two-tailed test) was considered statistically significant.

## Results

### Demographic and clinical characteristics of study subjects

Baseline demographic and clinical characteristics of study participants are summarized in **Table 1**. A total of 82 post-operative BA patients and 25 healthy controls were age- and gender-matched. As expected, liver stiffness, ALT, and AST values were significantly greater in BA patients than those in healthy controls (*P*<0.001).

**Table 1:** Baseline and clinical characteristics of healthy controls and BA patients.

Variables	Healthy controls (*n* = 25)	BA patients (*n* = 82)	*P*-value
**Age (years)**	10.00 (6.00–17.00)	10.00 (6.50–13.00)	0.271
**Gender (male:female)**	10:15	39:43	0.125
**BMI (kg/m^2^)**	18.06 (16.36–14.48)	16.05 (14.48–18.81)	0.085
**Liver stiffness (kPa)**	3.07 (3.15–4.55)	16.06 (8.00–40.10)	**<0.001**
**ALT (IU/L)**	9.00 (5.50–10.50)	93.00 (46.50–171.00)	**<0.001**
**AST (IU/L)**	26.00 (22.00–30.50)	101.00 (52.00–198.00)	**<0.001**
**TB (mg/dL)**	-	1.18 (0.47–3.78)	NA
**DB (mg/dL)**	-	0.68 (0.17–3.36)	NA
**ALP**	-	404.00 (273.75–552.50)	NA
**Albumin (g/dL)**	-	4.20 (3.70–4.60)	NA

Data are represented as either median with interquartile ranges (IQR, Q1-Q3) for continuous variables or frequency for categorical variables.

*P*-values marked with bold indicate statistically significant differences between the groups.

Abbreviations: ALP, alkaline phosphatase; ALT, alanine aminotransferase; AST, aspartate aminotransferase; BA, biliary atresia; BMI, body mass index; DB, direct bilirubin; TB, total bilirubin; NA, not available.

### Systemic cytokine profiles in healthy controls and BA patients

Descriptive data on systemic levels of cytokines in healthy controls and BA patients with different subgroups are displayed in **S1 Table**. Of 27 cytokines, 6 were inflammatory cytokines (IL-1β, IL-6, IL-7, IL-8, IL-9, and TNF-α), 5 were immunomodulatory cytokines (IL-2, IL-12p70, IL-15, IL-17, and IFN-γ), 6 were chemokines (eotaxin, IP-10, MCP-1, MIP-1α, MIP-1β, and RANTES), 5 were growth factors (G-CSF, GM-CSF, bFGF, PDGF, and VEGF), and 5 were anti-inflammatory cytokines (IL-1ra, IL-4, IL-5, IL-10, and IL-13).

#### Inflammatory cytokines

**Fig 1:**
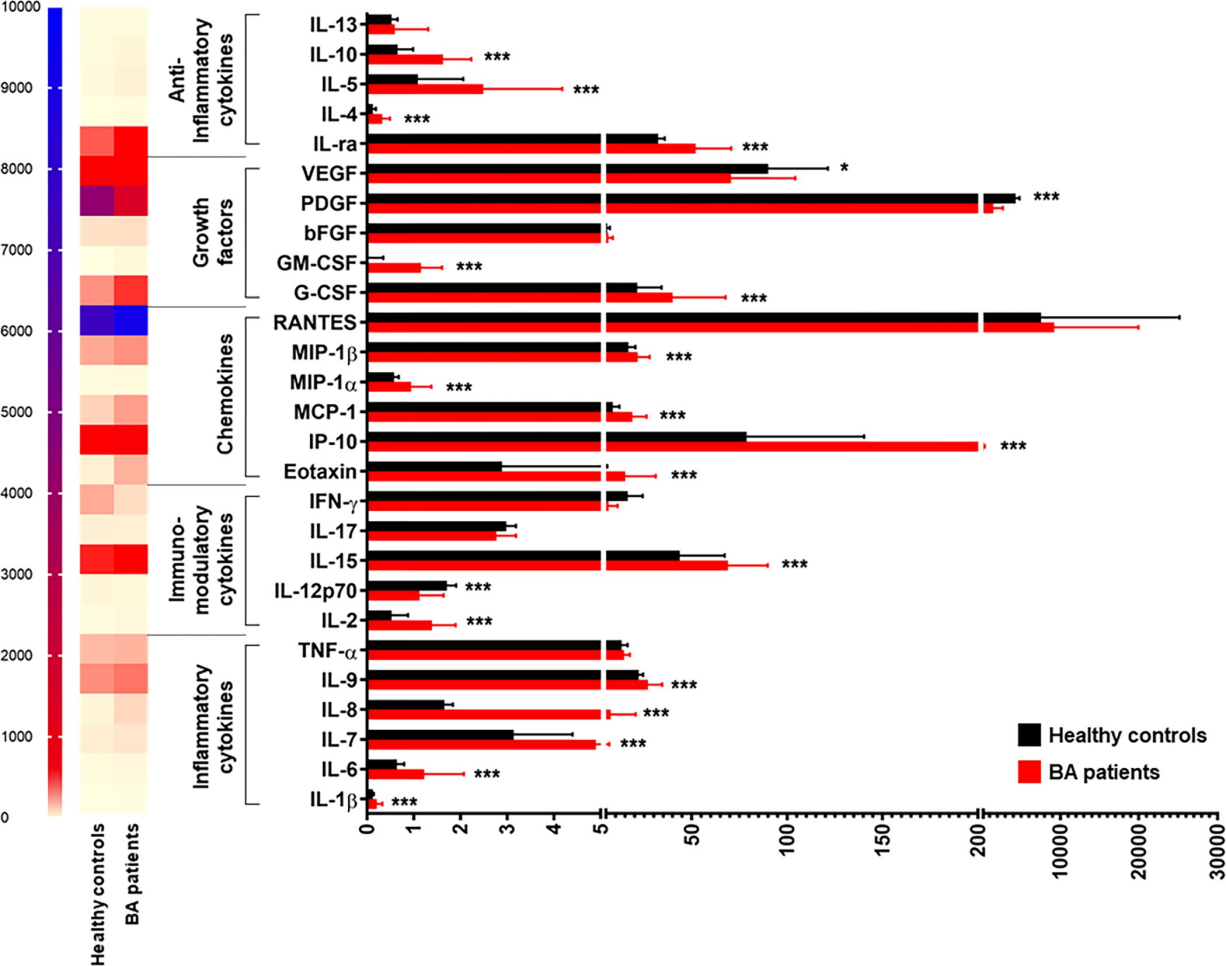
Systemic cytokine profiles in healthy controls and BA patients. *P<0.05, **P<0.01, and ***P<0.001. All *P*-values were calculated by Mann-Whitney *U* test.

Out of 6 inflammatory cytokines, 5 (IL-1β, IL-6, IL-7, IL-8, and IL-9) were significantly higher in the circulation of BA patients than those in healthy controls (*P*<0.001) (**Fig 1**). In contrast to these findings, there were no significant differences in plasma TNF-α levels between healthy controls and BA patients (**Fig 1**).

**Fig 2:**
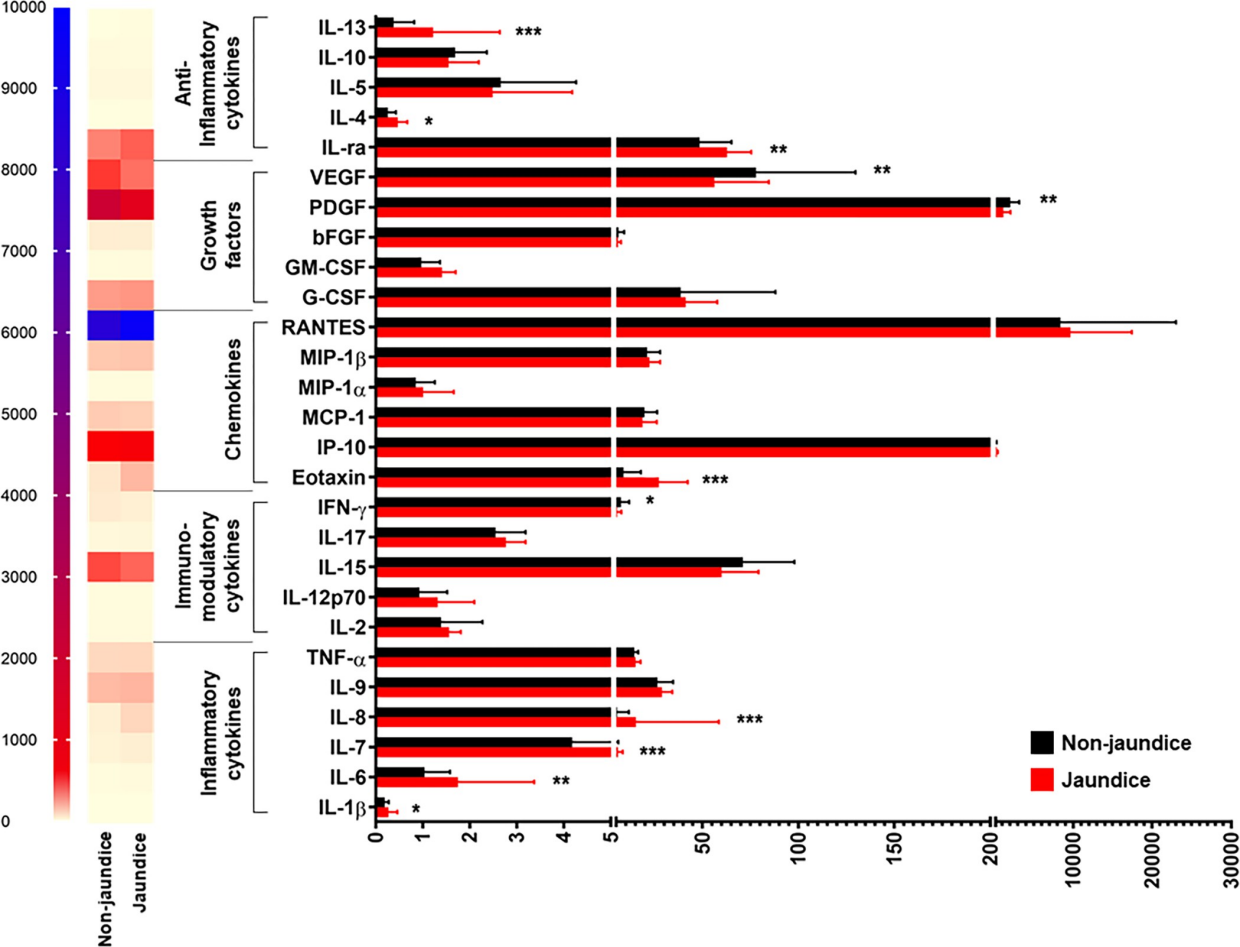
Systemic cytokine profiles in BA patients with and without jaundice. *P<0.05, **P<0.01, and ***P<0.001. All *P*-values were calculated by Mann-Whitney *U* test.

**Fig 3:**
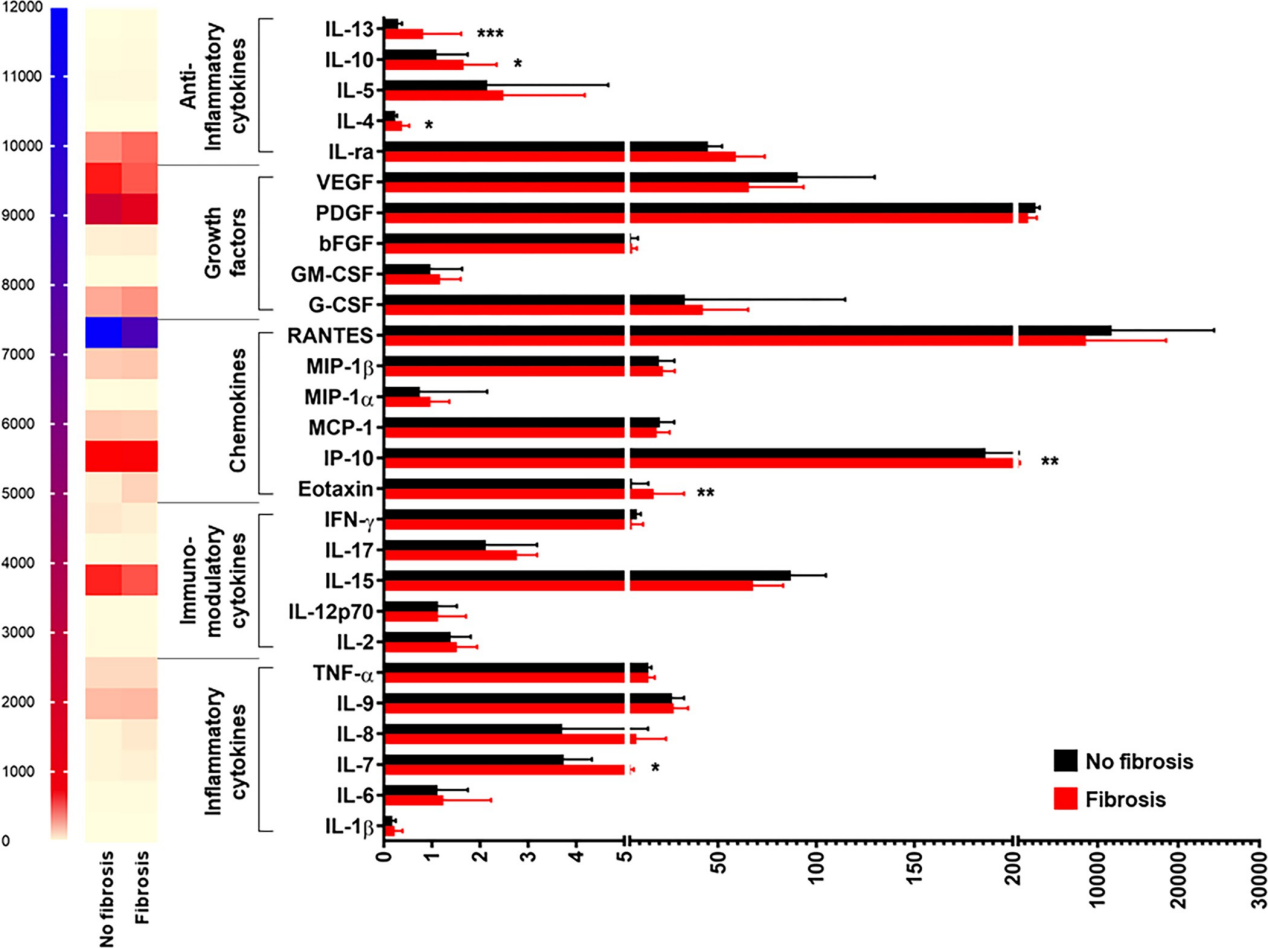
Systemic cytokine profiles in BA patients with and without fibrosis. *P<0.05, **P<0.01, and ***P<0.001. All *P*-values were calculated by Mann-Whitney *U* test.

**Fig 4:**
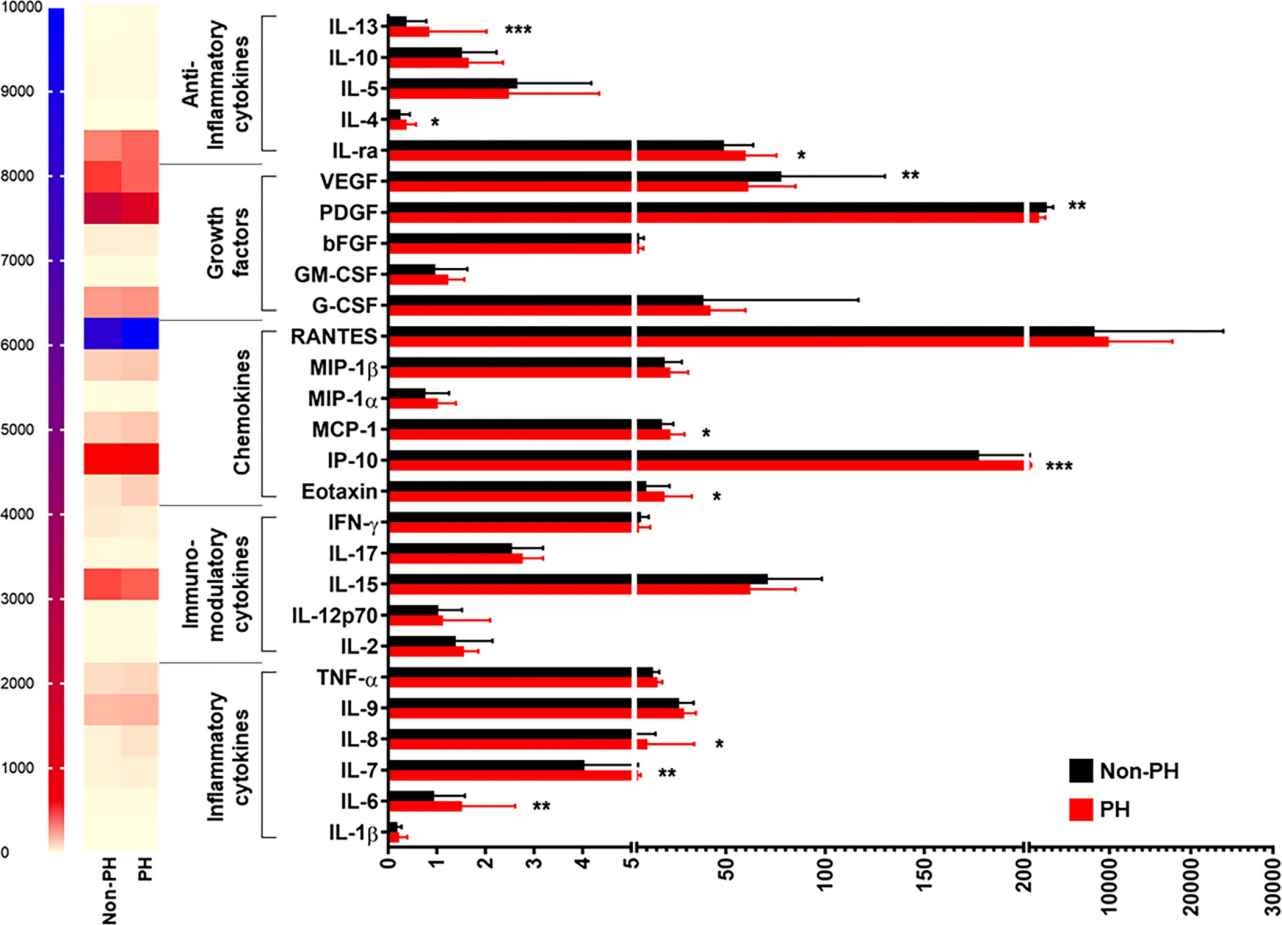
Systemic cytokine profiles in BA patients with and without portal hypertension. *P<0.05, **P<0.01, and ***P<0.001. All *P*-values were calculated by Mann-Whitney *U* test.

When disease severity was considered, stratified analysis by jaundice status showed that BA patients with jaundice had significantly increased plasma levels of 4 inflammatory cytokines including IL-1β (*P* = 0.010), IL-6 (*P* = 0.002), IL-7 (*P*<0.001), and IL-8 (*P*<0.001), compared with those without jaundice, as depicted in **Fig 2**. Instead, there were no significant differences in plasma levels of 2 inflammatory cytokines including IL-9 and TNF-α between the groups (**Fig 2**). Considering fibrosis status in BA patients, plasma IL-7 levels were found to be significantly higher in BA patients with fibrosis than those in non-fibrotic patients (*P* = 0.026), and no significant differences in plasma levels of remaining inflammatory cytokines including IL-1β, IL-6, IL-8, IL-9, and TNF-α between fibrotic and non-fibrotic groups were observed (**Fig 3**). In subsequent analysis based on PH status, BA patients with PH exhibited significantly elevated plasma levels of 3 inflammatory cytokines including IL-7 (*P* = 0.004), IL-6 (*P* = 0.009), and IL-8 (*P* = 0.014), compared with those without PH (**Fig 4**). Conversely, plasma levels of 3 inflammatory cytokines including IL-1β, IL-9, and TNF-α were not significantly different between those with and without PH (**Fig 4**).

#### Immunomodulatory cytokines

Among 5 immunomodulatory cytokines, plasma levels of 2 cytokines including IL-2 and IL-15 were found to be significantly greater in BA patients than those in healthy controls (*P*<0.001), while plasma levels of IL-12p70 and IFN-γ in BA patients were significantly lower than those in healthy controls (*P*<0.001) (**Fig 1**). Furthermore, no significant differences in plasma IL-17 levels between healthy controls and BA patients were detectable (**Fig 1**).

In stratified analysis by BA severity, compared with BA patients without jaundice, those with jaundice had significantly decreased plasma IFN-γ levels (*P* = 0.016), whereas there were no significant differences in plasma levels of 4 remaining cytokines including IL-2, IL-12p70, IL-15, and IL-17 between the groups (**Fig 2**). Based on fibrosis status, further analysis showed no significant differences in plasma levels of 5 immunomodulatory cytokines including IL-2, IL-12p70, IL-15, IL-17, and IFN-γ between fibrotic and non-fibrotic groups (**Fig 3**). Consistent with this, the differences in plasma levels of IL-2, IL-12p70, IL-15, IL-17, and IFN-γ between BA patients with PH and non-PH were not statistically significant (**Fig 4**).

#### Chemokines

Out of 6 chemokines, 5 (eotaxin, IP-10, MCP-1, MIP-1α, and MIP-1β) were significantly increased in the circulation of BA patients, compared to healthy controls (*P*<0.001) (**Fig 1**). On the other hand, no significant differences in plasma RANTES levels between healthy controls and BA patients were observed (**Fig 1**).

Compared with BA patients with non-jaundice, those with jaundice showed significant increases in plasma levels of 2 chemokines including eotaxin and IP-10 (*P* = 0.016, *P* = 0.003, respectively) (**Fig 2**). Contrary to this finding, there were no significant differences in plasma levels of 4 remaining chemokines including MCP-1, MIP-1α, MIP-1β, and RANTES between the groups (**Fig 2**). In stratified analysis by fibrosis status, significant elevations in plasma levels of 2 chemokines including eotaxin and IP-10 were found in BA patients with fibrosis, compared with those without fibrosis (*P* = 0.008, *P* = 0.008, respectively), while there were no significant differences in plasma levels of MCP-1, MIP-1α, MIP-1β, and RANTES between fibrotic and non-fibrotic groups (**Fig 3**). Additional analysis with regards to PH status uncovered that BA patients with PH showed significantly lower plasma levels of 3 chemokines including eotaxin (*P* = 0.001), IP-10 (*P*<0.001), and MCP-1 (*P* = 0.023) than those with non-PH (**Fig 4**). Despite alterations in plasma levels of 3 remaining chemokines including MIP-1α, MIP-β, and RANTES between non-PH and PH cases, the differences were not statistically significant (**Fig 4**).

#### Growth factors

Of 5 growth factors, 2 (G-CSF and GM-CSF) were significantly higher in the circulation of BA patients than those in healthy controls (*P*<0.001) (**Fig 1**). Conversely, 2 out of 5 growth factors including PDGF and VEGF were significantly lower in the circulation in BA patients than those in healthy controls (*P*<0.001, *P* = 0.032, respectively), while there were no significant differences in plasma bFGF levels between healthy controls and BA patients (**Fig 1**).

In addition to the above findings, significant decreases in plasma levels of 2 growth factors including PDGF (*P* = 0.007) and VEGF (*P* = 0.003) were found in jaundice cases, compared with non-jaundice cases (**Fig 2**). Furthermore, there were no significant differences in plasma levels of 3 remaining growth factors including bFGF, G-CSF, and GM-CSF between the groups (**Fig 2**). Further analysis by fibrosis status demonstrated no significant differences in plasma levels of all growth factors including bFGF, G-CSF, GM-CSF, PDGF, and VEGF between fibrotic and non-fibrotic groups (**Fig 3**). Compared with BA patients with non-PH, the patients with PH showed significantly increased plasma VEGF levels (*P* = 0.025) and no significant differences in plasma levels of 4 growth factors including bFGF, G-CSF, GM-CSF, and PDGF (**Fig 4**).

#### Anti-inflammatory cytokines

Compared to healthy controls, 4 out of 5 anti-inflammatory cytokines including IL-1ra, IL-4, IL-5, and IL-10 were significantly elevated in BA patients (*P*<0.001) (**Fig 1**). However, differences in plasma IL-13 levels between BA patients and healthy controls were not statistically significant (**Fig 1**).

Compared with BA patients with non-jaundice, the patients with jaundice had significantly increased plasma levels of 3 anti-inflammatory cytokines including IL-ra (*P* = 0.002), IL-4 (*P* = 0.010), and IL-13 (*P*<0.001) (**Fig 2**). Furthermore, there were no significant differences in plasma levels of 2 anti-inflammatory cytokines including IL-5 and IL-10 between jaundice and non-jaundice groups (**Fig 2**). Compared with BA patients without fibrosis, plasma levels of 3 anti-inflammatory cytokines including IL-4 (*P* = 0.026), IL-10 (*P* = 0.048), and IL-13 (*P*<0.001) were significantly increased in those with fibrosis (**Fig 3**). Nevertheless, there were no significant differences in plasma levels of IL-1ra and IL-5 between fibrotic and non-fibrotic patients (**Fig 3**). Compared with BA patients without PH, significant increases in plasma levels of 3 anti-inflammatory cytokines including IL-1ra (*P* = 0.043), IL-4 (*P* = 0.017), and IL-13 (*P*<0.001) were observed in BA patients with PH (**Fig 4**). Contrariwise, plasma levels of 2 remaining anti-inflammatory cytokines including IL-5 and IL-10 between non-PH and PH groups were not significantly different (**Fig 4**).

### Associations between unfavorable outcomes and systemic cytokine profiles in BA patients

Because age, gender, and BMI may be potential confounding factors affecting BA outcomes, multivariable logistic regression was conducted to determine associations of BA and its unfavorable outcomes including jaundice, significant fibrosis, and PH with systemic cytokine profiles. As demonstrated in **Table 2**, after adjusting for the above confounders, increased plasma levels of 17 out of 27 cytokines including inflammatory cytokines [IL-1β (OR = 1.04, 95% CI: 1.02, 1.06, *P* = 0.001), IL-6 (OR = 1.01, 95% CI: 1.00, 1.01, *P* = 0.001), IL-7 (OR = 1.75, 95% CI: 1.22, 2.50, *P* = 0.002), IL-8 (OR = 3.30, 95% CI: 1.45, 7.50, *P* = 0.004), and IL-9 (OR = 1.26, 95% CI: 1.10, 1.45, *P* = 0.001], immunomodulatory cytokines [IL-2 (OR = 1.08, 95% CI: 1.06, 1.18, *P*<0.001) and IL-15 (OR = 1.06, 95% CI: 0.76, 1.45, *P* = 0.001)], chemokines [eotaxin (OR = 1.23, 95% CI: 1.09, 1.39, *P* = 0.001), IP-10 (OR = 1.03, 95% CI: 1.01, 1.04, *P* = 0.001), MCP-1 (OR = 1.48, 95% CI: 1.19, 1.84, *P* = 0.001), MIP-1α (OR = 17.25, 95% CI: 2.20, 135.05, *P* = 0.007), and MIP-1β (OR = 1.13, 95% CI: 1.01, 1.26, *P* = 0.027)], growth factor [G-CSF (OR = 1.08, 95% CI: 1.02, 1.13, *P* = 0.008)], and anti-inflammatory cytokines [IL-1ra (OR = 1.13, 95% CI: 1.05, 1.21, *P* = 0.001), IL-4 (OR = 1.02, 95% CI: 1.01, 1.03, *P*<0.001), IL-5 (OR = 2.64, 95% CI: 1.43, 4.85, *P* = 0.002), and IL-10 (OR = 1.09, 95% CI: 1.01, 1.20, *P*<0.001)] were found to be significantly associated with BA. On the other hand, decreased plasma levels of 2 out of 27 cytokines including IFN-γ (OR = 0.87, 95% CI: 0.80, 0.94, *P* = 0.001) and PDGF (OR = 0.88, 95% CI: 0.82, 0.94, *P*<0.001) were significantly associated with BA.

**Table 2:** Multivariate logistic regression analyses showing associations of BA and its unfavorable outcomes including jaundice, significant fibrosis, and PH with systemic cytokine profiles.

	**BA**		**Jaundice**		**Fibrosis**		**PH**	
	^**a**^ **OR (95% CI)**	***P*-value**	^**a**^ **OR (95% CI)**	***P*-value**	^**a**^ **OR (95% CI)**	***P*-value**	^**a**^ **OR (95% CI)**	***P*-value**
**Inflammatory cytokines**								
IL-1β	1.04 (1.02, 1.06)	**0.001**	1.00 (0.99, 1.00)	0.508	1.00 (0.99, 1.00)	0.247	1.00 (0.99, 1.00)	0.508
IL-6	1.01 (1.00, 1.01)	**0.001**	0.95 (0.51, 1.76)	0.868	0.96 (0.86, 1.08)	0.512	1.05 (0.93, 1.20)	0.433
IL-7	1.75 (1.22, 2.50)	**0.002**	1.42 (1.14, 1.76)	**0.002**	1.54 (1.11, 2.12)	**0.010**	1.19 (0.99, 1.44)	0.061
IL-8	3.30 (1.45, 7.50)	**0.004**	0.98 (0.95, 1.02)	0.354	0.99 (0.99, 1.00)	0.234	1.00 (0.99, 1.00)	0.452
IL-9	1.26 (1.10, 1.45)	**0.001**	1.01 (0.98, 1.06)	0.606	1.04 (0.97, 1.12)	0.304	1.01 (0.97, 1.05)	0.670
TNF-α	1.11 (0.97, 1.28)	0.132	1.02 (0.94, 1.12)	0.629	1.03 (0.93, 1.15)	0.568	1.04 (0.95, 1.22)	0.407
**Immunomodulatory cytokines**								
IL-2	1.08 (1.06, 1.18)	**<0.001**	1.03 (0.64, 1.66)	0.909	1.07 (0.68, 1.70)	0.765	0.93 (0.67, 1.30)	0.678
IL-12p70	0.75 (0.47, 1.20)	0.232	1.24 (0.87, 1.77)	0.240	1.21 (0.70, 2.10)	0.495	1.17 (0.81, 1.68)	0.415
IL-15	1.06 (1.02, 1.09)	**0.001**	0.99 (0.98, 1.01)	0.256	0.99 (0.98, 1.01)	0.405	0.99 (0.97, 1.00)	0.110
IL-17	1.05 (0.76, 1.45)	0.750	1.08 (0.91, 1.29)	0.374	1.60 (0.86, 2.99)	0.138	1.27 (0.93, 1.74)	0.140
IFN-γ	0.87 (0.80, 0.94)	**0.001**	0.94 (0.87, 1.01)	0.087	1.01 (0.95, 1.07)	0.824	0.98 (0.93, 1.03)	0.438
**Chemokines**								
Eotaxin	1.23 (1.09, 1.39)	**0.001**	1.09 (1.05, 1.14)	**<0.001**	1.09 (1.02, 1.61)	**0.010**	1.05 (1.01, 1.08)	**0.008**
IP-10	1.03 (1.01, 1.04)	**0.001**	1.00 (1.00, 1.01)	**0.008**	1.01 (1.00, 1.01)	**0.027**	1.01 (1.00, 1.01)	**0.002**
MCP-1	1.48 (1.19, 1.84)	**0.001**	1.00 (0.99, 1.01)	0.900	0.99 (0.97, 1.01)	0.195	1.00 (0.99, 1.01)	0.943
MIP-1α	17.25 (2.20, 135.05)	**0.007**	0.96 (0.88, 1.05)	0.356	0.96 (0.91, 1.02)	0.210	0.97 (0.91, 1.04)	0.379
MIP-1β	1.13 (1.01, 1.26)	**0.027**	0.99 (0.96, 1.02)	0.349	0.98 (0.96, 1.01)	0.260	1.00 (0.98, 1.03)	0.971
RANTES	0.99 (0.95, 1.04)	0.675	1.00 (0.99, 1.00)	0.612	1.00 (0.99, 1.00)	0.776	1.00 (0.99, 1.00)	0.536
**Growth factors**								
G-CSF	1.08 (1.02, 1.13)	**0.008**	1.00 (0.99, 1.00)	0.220	1.00 (0.99, 1.01)	0.941	0.99 (0.98, 1.00)	0.084
GM-CSF	1.05 (0.84, 1.31)	0.697	1.59 (0.93, 2.73)	0.091	1.64 (0.68, 3.99)	0.271	1.00 (0.71, 1.43)	0.983
bFGF	1.10 (0.87, 1.38)	0.433	0.91 (0.78, 1.06)	0.237	1.07 (0.88, 1.30)	0.505	0.97 (0.84, 1.12)	0.660
PDGF	0.88 (0.82, 0.94)	**<0.001**	0.99 (0.99, 1.00)	**0.012**	0.99 (0.99, 1.00)	0.648	1.00 (0.99, 1.00)	0.366
VEGF	0.95 (0.91, 1.03)	0.265	0.99 (0.97, 1.00)	**0.029**	1.00 (0.99, 1.01)	0.816	0.99 (0.98, 1.00)	0.084
**Anti-inflammatory cytokines**								
IL-1ra	1.13 (1.05, 1.21)	**0.001**	1.02 (1.00, 1.04)	**0.033**	1.02 (0.99, 1.05)	0.111	1.02 (1.00, 1.03)	0.091
IL-4	1.02 (1.01, 1.03)	**<0.001**	1.00 (1.00, 1.01)	**0.009**	1.00 (1.00, 1.01)	0.072	1.21 (0.41, 3.62)	0.729
IL-5	2.64 (1.43, 4.85)	**0.002**	1.00 (0.83, 1.21)	0.998	1.08 (0.83, 1.39)	0.584	0.93 (0.77, 1.12)	0.427
IL-10	1.09 (1.01, 1.20)	**<0.001**	0.96 (0.80, 1.15)	0.660	1.37 (0.75, 2.52)	0.310	0.92 (0.78, 1.10)	0.378
IL-13	1.05 (0.76, 1.45)	0.750	1.96 (1.19, 3.24)	**0.009**	51.26 (2.84, 924.74)	**0.008**	1.27 (0.93, 1.74)	0.140

*P*-values marked with bold indicate statistically significant differences between 2 groups (BA *vs* healthy controls, jaundice *vs* non-jaundice, fibrosis *vs* no fibrosis, PH *vs* non-PH).

^a^OR was adjusted for age, gender, and BMI.

Abbreviations: BA, biliary atresia; bFGF, basic fibroblast growth factor; G-CSF, granulocyte colony stimulating factor; GM-CSF, granulocyte macrophage colony-stimulating factor; IFN, interferon; IL, interleukin; IL-1ra, anti-inflammatory cytokines including IL-1 receptor antagonist; IP, IFN-γ-induced protein; MCP, monocyte chemoattractant protein; MIP, macrophage inflammatory protein; PDGF, platelet-derived growth factor; PH, portal hypertension; RANTES, Regulated on Activation, Normal T Expressed and Secreted; TNF, tumor necrosis factor; VEGF, vascular endothelial growth factor.

Considering unfavorable outcomes of post-operative BA patients including jaundice, significant fibrosis, and PH, multivariable logistic regression analysis with adjusting for age, gender, and BMI showed that in BA patients, jaundice was significantly associated with elevated plasma levels of IL-7 (OR = 1.42, 95% CI: 1.14, 1.76, *P* = 0.002), eotaxin (OR = 1.09, 95% CI: 1.05, 1.14, *P<*0.001), IP-10 (OR = 1.05, 95% CI: 1.01, 1.08, *P* = 0.008), IL-1ra (OR = 1.02, 95% CI: 1.00, 1.04, *P* = 0.033), IL-4 (OR = 1.04, 95% CI: 1.01, 1.08, *P* = 0.009), and IL-13 (OR = 1.96, 95% CI: 1.19, 3.24, *P* = 0.009), but significantly associated with reduced plasma levels of PDGF (OR = 0.99, 95% CI: 0.99, 1.00, *P* = 0.012) and VEGF (OR = 0.99, 95% CI: 1.19, 3.24, *P* = 0.029). Consistent with this, significant fibrosis in BA patients was found to be significantly associated with increased plasma levels of IL-7 (OR = 1.54, 95% CI: 1.11, 2.12, *P* = 0.010), eotaxin (OR = 1.09, 95% CI: 1.02, 1.61, *P* = 0.010), IP-10 (OR = 1.01, 95% CI: 1.00, 1.01, *P* = 0.027), and IL-13 (OR = 51.26, 95% CI: 2.84, 924.74, *P* = 0.008). Portal hypertension in BA patients was also detected to be significantly associated with elevated plasma levels of eotaxin (OR = 1.05, 95% CI: 1.01, 1.08, *P* = 0.008) and IP-10 (OR = 1.01, 95% CI: 1.01, 1.02, *P* = 0.008).

### Associations between clinical parameters and systemic cytokine profiles in BA patients

**Fig 5:**
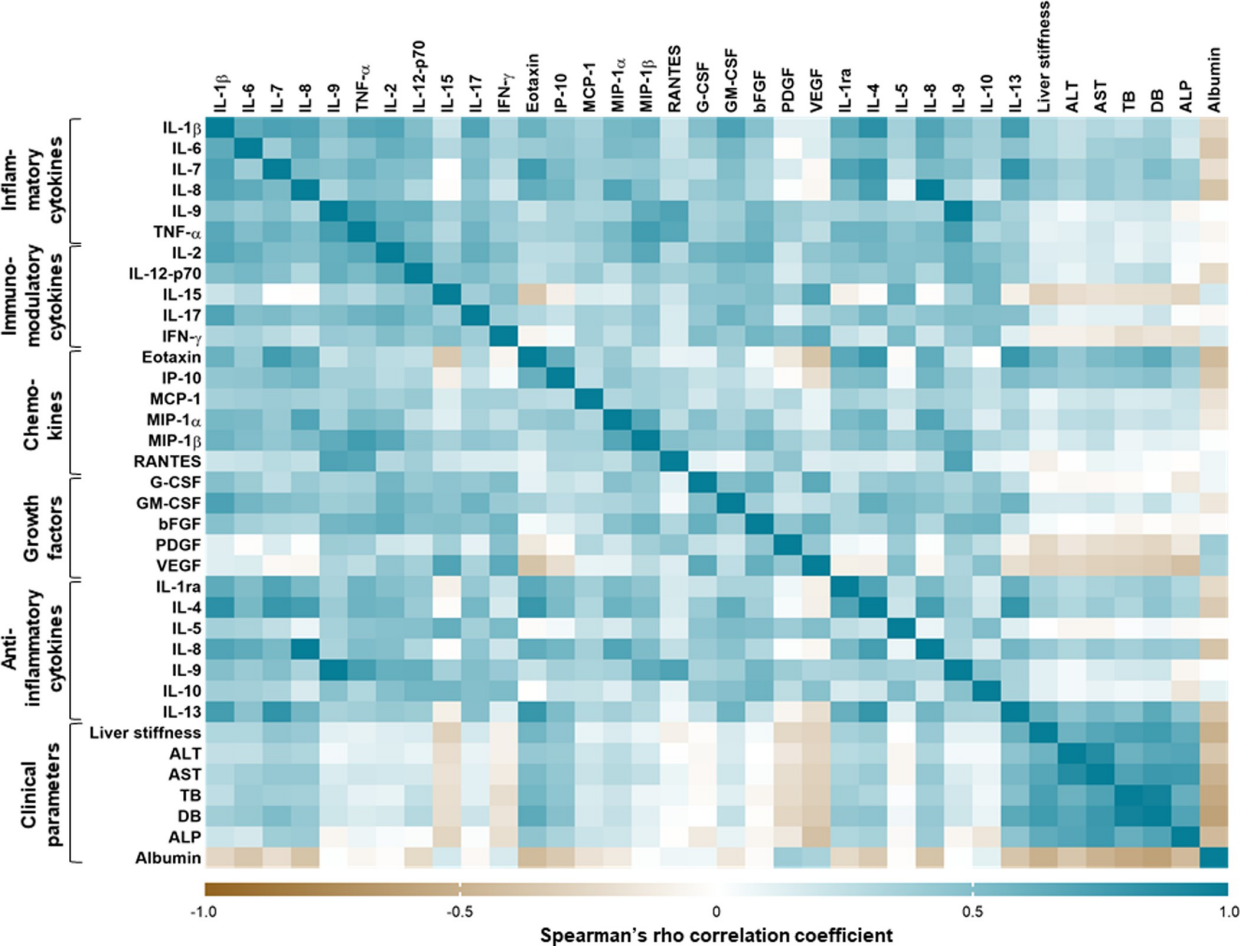
Heatmap of Spearman’s Rho correlations between systemic cytokine profiles and clinical parameters in BA patients.

Associations between clinical parameters and systemic cytokine profiles in BA patients are represented as a matrix heatmap in **Fig 5**. Spearman’s rho correlation analysis uncovered that liver stiffness values were positively associated with plasma levels of 18 cytokines including 4 inflammatory cytokines [IL-1β (*r* = 0.56, *P*<0.001), IL-6 (*r* = 0.53, *P*<0.001), IL-7 (*r* = 0.51, *P*<0.001), and IL-8 (*r* = 0.67, *P*<0.001)], 1 immunomodulatory cytokine [IL-2 (*r* = 0.47, *P*<0.001)], 5 chemokines [eotaxin (*r* = 0.63, *P*<0.001), IP-10 (*r* = 0.67, *P*<0.001), MCP-1 (*r* = 0.43, *P*<0.001), MIP-1α (*r* = 0.38, *P*<0.001), and MIP-1β (*r* = 028, *P* = 0.004)], 2 growth factors [G-CSF (*r* = 0.31, *P* = 0.001) and GM-CSF (*r* = 0.40, *P*<0.001)], as well as 5 anti-inflammatory cytokines [IL-1ra (*r* = 0.52, *P*<0.001), IL-4 (*r* = 0.58, *P*<0.001), IL-5 (*r* = 0.23, *P* = 0.016), IL-10 (*r* = 0.47, *P*<0.001), and IL-13 (*r* = 0.49, *P*<0.001)], but negatively associated with plasma levels of 3 cytokines including 1 chemokine [IFN-γ (*r* = -0.36, *P*<0.001)] and 2 growth factors [PDGF (*r* = -0.55, *P*<0.001) and VEGF (*r* = -0.55, *P*<0.001)].

Somewhat similar to liver stiffness, serum aminotransferases including ALT and AST were detected to be positively correlated with 17 cytokines including 5 inflammatory cytokines [IL-1β (*r* = 0.53, *P*<0.001; *r* = 0.54, *P*<0.001; respectively), IL-6 (*r* = 0.50, *P*<0.001; *r* = 0.56, *P*<0.001; respectively), IL-7 (*r* = 0.50, *P*<0.001; *r* = 0.56, *P*<0.001; respectively), IL-8 (*r* = 0.64, *P*<0.001; *r* = 0.67, *P*<0.001; respectively), and IL-9 (*r* = 0.32, *P* = 0.001; *r* = 0.67, *P*<0.001; respectively)], 1 immunomodulatory cytokine [IL-2 (*r* = 0.49, *P*<0.001; *r* = 0.49, *P*<0.001; respectively)], 5 chemokines [eotaxin (*r* = 0.64, *P*<0.001; *r* = 0.59, *P*<0.001; respectively), IP-10 (*r* = 0.66, *P*<0.001; *r* = 0.63, *P*<0.001; respectively), MCP-1 (*r* = 0.53, *P*<0.001; *r* = 0.50, *P*<0.001; respectively), MIP-1α (*r* = 0.46, *P*<0.001; *r* = 0.46, *P*<0.001; respectively), and MIP-1β (*r* = 0.33, *P* = 0.001; *r* = 0.37, *P*<0.001; respectively)], 2 growth factors [G-CSF (*r* = 0.25, *P* = 0.010; *r* = 0.30, *P* = 0.001; respectively) and GM-CSF (*r* = 0.42, *P*<0.001; *r* = 0.41, *P*<0.001; respectively)], as well as 4 anti-inflammatory cytokines [IL-1ra (*r* = 0.51, *P*<0.001; *r* = 0.53, *P*<0.001; respectively), IL-4 (*r* = 0.57, *P*<0.001; *r* = 0.61, *P*<0.001; respectively), IL-10 (*r* = 0.45, *P*<0.001; *r* = 0.44, *P*<0.001; respectively), and IL-13 (*r* = 0.46, *P*<0.001; *r* = 0.37, *P*<0.001; respectively)], but inversely correlated with plasma levels of 3 cytokines including 1 chemokine [IFN-α (*r* = -0.35, *P* = 0.001; *r* = 0.31, *P*<0.001; respectively)], 2 growth factors [PDGF (*r* = -0.52, *P*<0.001; *r* = 0.51, *P*<0.001; respectively) and VEGF (*r* = -0.38, *P*<0.001; *r* = 0.31, *P*<0.001; respectively)].

Apart from the above findings, serum bilirubin levels consisting of total bilirubin and direct bilirubin were found to be directly associated with plasma levels of 10 cytokines including 4 inflammatory cytokines [IL-1β (*r* = 0.26, *P* = 0.020; *r* = 0.37, *P* = 0.001; respectively), IL-6 (*r* = 0.40, *P*<0.001; *r* = 0.42, *P*<0.001; respectively), IL-7 (*r* = 0.37, *P* = 0.001; *r* = 0.52, *P*<0.001; respectively), and IL-8 (*r* = 0.36, *P* = 0.001; *r* = 0.42, *P*<0.001; respectively)], 1 immunomodulatory cytokine [IL-12p70 (*r* = 0.24, *P* = 0.031; *r* = 0.29, *P* = 0.009; respectively)], 2 chemokines [eotaxin (*r* = 0.56, *P*<0.001; *r* = 0.65, *P*<0.001; respectively) and IP-10 (*r* = 0.45, *P*<0.001; *r* = 0.48, *P*<0.001; respectively)], as well as 3 anti-inflammatory cytokines [IL-1ra (*r* = 0.38, *P* = 0.001; *r* = 0.44, *P*<0.001; respectively), IL-4 (*r* = 0.32, *P* = 0.003; *r* = 0.45, *P*<0.001; respectively), and IL-13 (*r* = 0.49, *P*<0.001; *r* = 0.66, *P*<0.001; respectively)], but negatively associated with plasma levels of 2 growth factors [PDGF (*r* = -0.23, *P* = 0.037; *r* = -0.27, *P* = 0.016; respectively) and VEGF (*r* = -0.31, *P*<0.001; *r* = 0.34, *P*<0.001; respectively)].

Subsequent analysis showed that ALP levels were closely related to plasma levels of 7 cytokines including 2 inflammatory cytokines [IL-7 (*r* = 0.38, *P* = 0.001) and IL-8 (*r* = 0.40, *P*<0.001)], 2 chemokines [eotaxin (*r* = 0.45, *P*<0.001) and IP-10 (*r* = 0.34, *P* = 0.003)], as well as 3 anti-inflammatory cytokines [IL-1ra (*r* = 0.32, *P* = 0.005), IL-4 (*r* = 0.35, *P* = 0.002), and IL-13 (*r* = 0.46, *P*<0.001)], but negatively correlated with plasma levels of immunumodulatory cytokine [IL-15 (*r* = -0.28, *P* = 0.015)] and growth factor [VEGF (*r* = -0.41, *P*<0.001)].

In contrast to all above-mentioned correlations, serum albumin was found to be inversely associated with plasma levels of 7 cytokines including 3 inflammatory cytokines [IL-1β (*r* = -0.27, *P* = 0.029), IL-6 (*r* = -0.36, *P* = 0.002), and IL-8 (*r* = -0.38, *P* = 0.001)], 2 chemokines [eotaxin (*r* = -0.44, *P*<0.001) and IP-10 (*r* = -0.35, *P* = 0.004)], as well as 3 anti-inflammatory cytokines [IL-1ra (*r* = -0.25, *P* = 0.004), IL-4 (*r* = -0.34, *P* = 0.005), and IL-13 (*r* = -0.38, *P* = 0.002)], but positively associated with plasma levels of 2 growth factors including PDGF (*r* = 0.39, *P*<0.001) and VEGF (*r* = 0.34, *P* = 0.005).

### Systemic cytokines as potential biomarkers for BA

**Fig 6:**
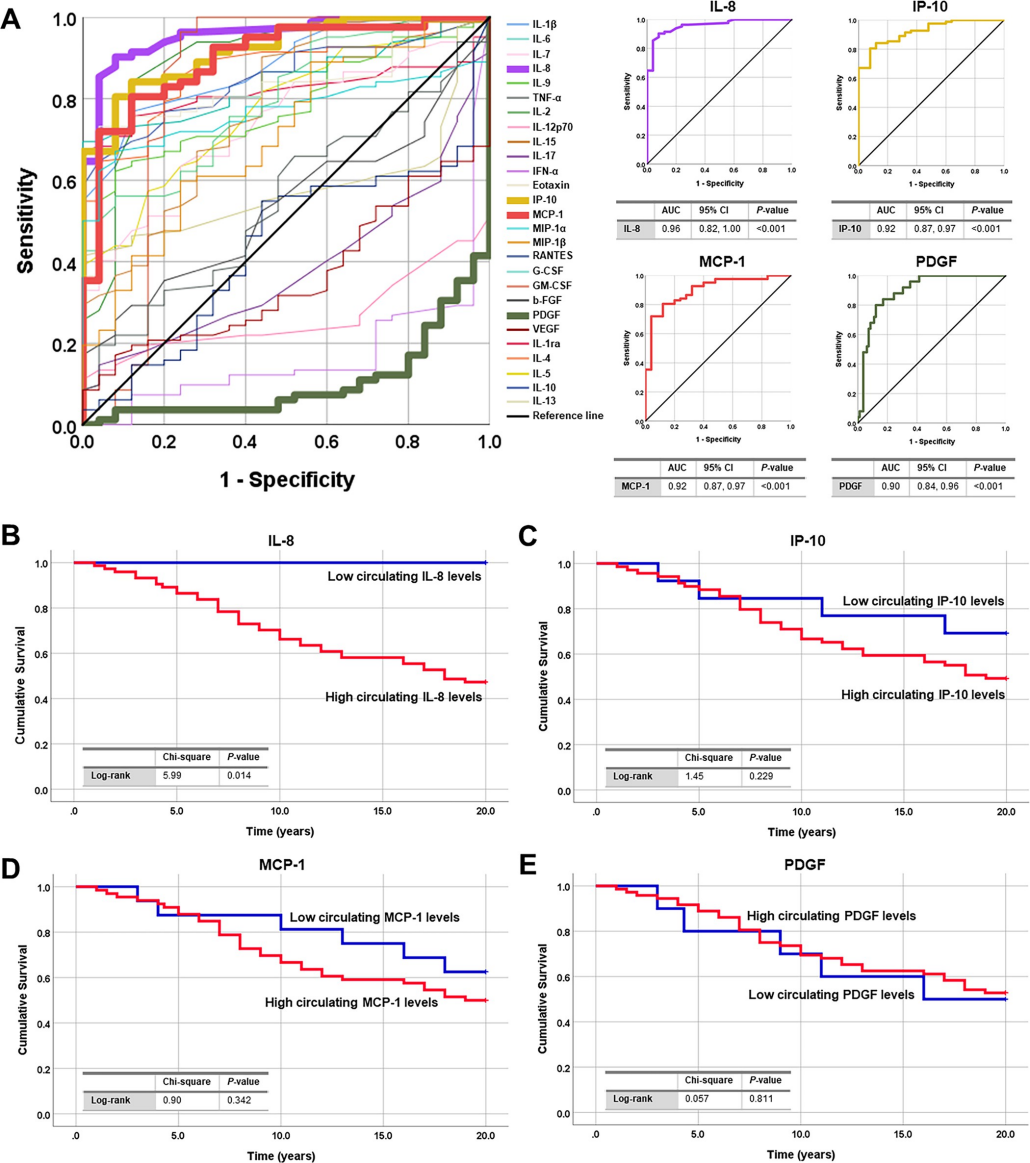
Kaplan-Meier survival curve of post-operative BA patients and receiver operating characteristic curve demonstrating diagnostic values of systemic cytokines in BA patients. (A) Possibility of systemic cytokines as biomarkers for distinguishing BA patients from healthy controls. (A) Comparing survival curves between BA patients with low and high plasma IL-8 levels. (B) Comparing survival curves between BA patients with low and high plasma IP-10 levels. (D) Comparing survival curves between BA patients with low and high plasma MCP-1 levels. (E) Comparing survival curves between BA patients with low and high plasma PDGF levels.

The ROC curve was constructed to calculate the area under the ROC curve (AUC) used for identifying the potential usefulness of systemic cytokines as biomarkers for BA. Based on ROC curve analysis as displayed in **Fig 6A**, among 27 systemic cytokines, 4 (IL-8, IP-10, MCP-1, and PDGF) yielded an AUC of more than 0.90, thereby establishing their potential as more sensitive and specific biomarkers for differentiating BA patients from healthy controls than others. The optimal cutoff values of those cytokines were defined at 2.29 pg/mL (IL-8), 150.48 pg/mL (IP-10), 12.92 pg/mL (MCP-1), and 3,263.33 pg/mL (PDGF), respectively, which provided an AUC of 0.96 (IL-8, 95% CI: 0.82, 1.00; *P*<0.001), 0.92 (IP-10, 95% CI: 0.87, 0.97; *P*<0.001), 0.92 (MCP-1, 95% CI: 0.87, 0.97; *P*<0.001), and 0.90 (PDGF, 95% CI: 0.84, 0.96; *P*<0.001), respectively, a sensitivity of 90.2% (IL-8), 84.1% (IP-10), 80.5% (MCP-1), and 80.0% (PDGF) respectively, and a specificity of 92.0% (IL-8), 88.0% (IP-10), 88.0% (MCP-1), and 87.8% (PDGF), respectively.

### Association between increased plasma IL-8 and a decreased survival rate of BA patients

Given significant alterations in plasma levels of cytokines in BA patients–especially in those with unfavorable outcomes, Kaplan-Meier survival analysis was executed to evaluate the possible effect of systemic cytokines on survival rate in BA patients. Using the optimal cutoff value derived from ROC curve analysis, 4 candidate cytokines were categorized into 2 groups: low plasma levels (IL-8 <2.29 pg/mL, *n* = 8; IP-10 <150.48 pg/mL, *n* = 13; MCP-1 <12.92 pg/mL, *n* = 16; and PDGF <3,263.33 pg/mL, *n* = 72) and high plasma levels (IL-8 ≥2.29 pg/mL, *n* = 73; IP-10 ≥150.48 pg/mL, *n* = 69; MCP-1 ≥12.92 pg/mL, *n* = 66; and PDGF ≥3,263.33 pg/mL, *n* = 10). From those candidate cytokines, BA patients with high plasma IL-8 levels had a significantly reduced survival rate, compared with the patients with low plasma IL-8 levels (Log-rank: χ^2^ = 5.99, *P* = 0.014) (**Fig 6B**). Nonetheless, there were no significant associations between plasma levels of 3 remaining cytokines including IP-10, MCP-1, as well as PDGF and a survival rate of BA patients following Kasai operation (**Fig 6C–6E**).

### Relative mRNA expressions of candidate cytokines in BA livers

**Fig 7:**
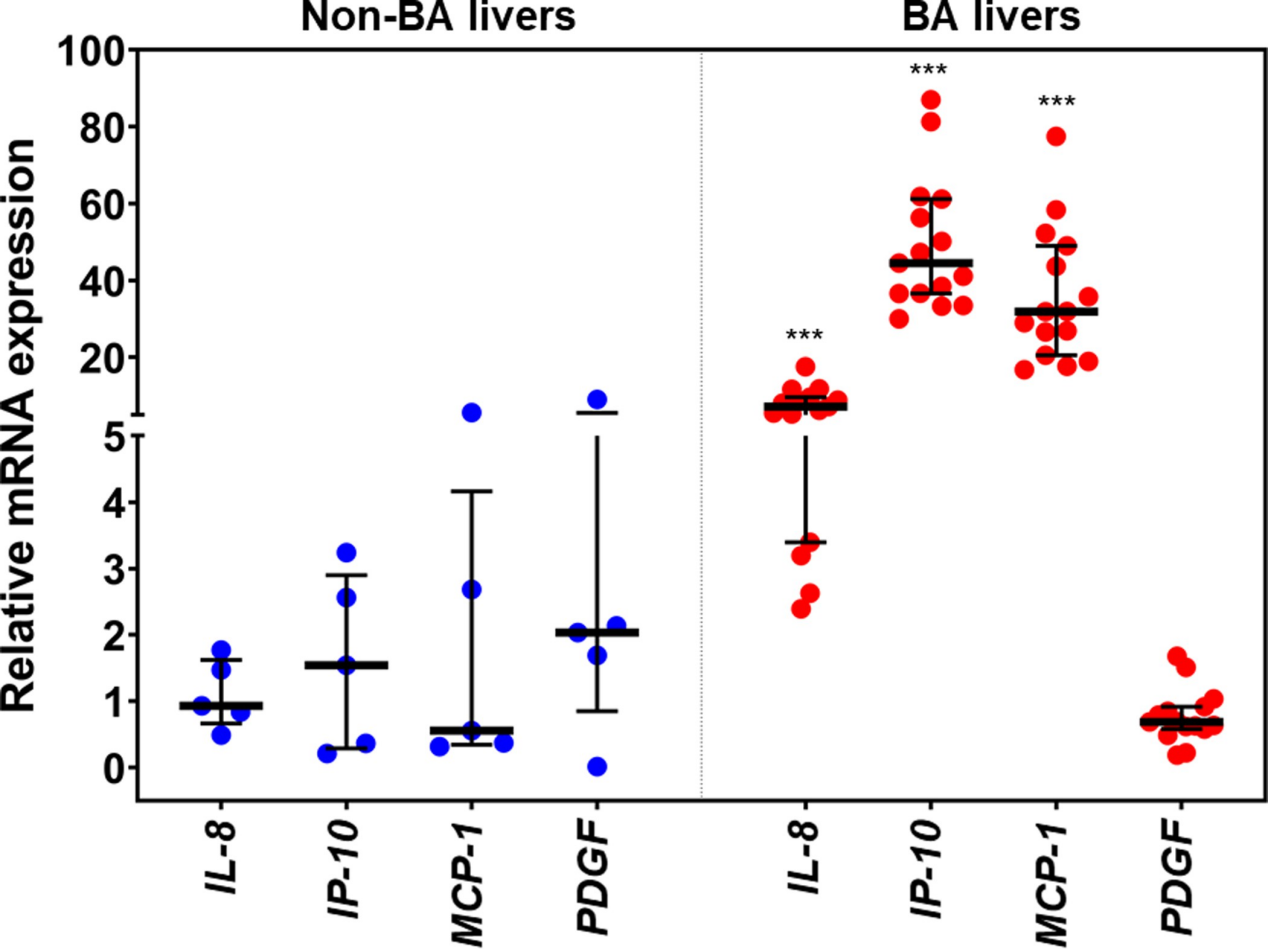
Relative mRNA expressions of cytokines including *IL-8*, *IP-10*, *MCP-1*, and *PDGF* in non-BA livers and BA livers. *P<0.05, **P<0.01, and ***P<0.001. All *P*-values were calculated by Mann-Whitney *U* test.

Based on ROC curve analysis, 4 out of 27 cytokines, which yielded the AUC of more than 0.90, were selected to determine their mRNA expressions in 20 BA livers and 5 non-BA livers. Quantitative real-time PCR analysis revealed that relative mRNA expressions of *IL-8*, *IP-10*, and *MCP-1* were significantly up-regulated in BA livers, compared with non-BA (*P* = 0001) (**Fig 7)**. In contrary to this, although relative *PDGF* mRNA expression was found to be lower in BA livers than that in non-BA livers, the difference was not statistically significant.

## Discussion

Despite breakthroughs in our understanding of critical aspects relevant to BA etiology, the disease progresses to end-stage liver failure in most BA patients and is the most common indication of pediatric liver transplantation, due to the lack of reliable non-invasive diagnostic biomarkers. It is important to note that a timely diagnosis of BA and non-invasive diagnostic techniques are both essential for optimal patients’ outcomes following KPE, for which a greater understanding of mechanisms associated with biliary injury and fibrosis will help to identify novel specific biomarkers of BA. In the present study, we therefore investigated profiles of systemic cytokines including inflammatory cytokines, immunomodulatory cytokines, chemokines, growth factors, and anti-inflammatory cytokines in post-operative BA patients and found significant alterations in plasma levels of those cytokines in BA patients–especially those with advanced-stage including jaundice, fibrosis, and PH. These findings support the notion that inflammation and immune dysregulation may be implicated in biliary duct injury-induced chronic liver failure in BA patients, which may open the door to discovery of specific biomarkers for monitoring unfavorable outcomes in post-operative BA.

Given that cytokines released from immune cells play key roles in regulation of immune and inflammatory responses, production of numerous cytokines including inflammatory cytokines and chemokines from neonatal liver B cells of murine BA has been shown to induce biliary duct injury via activation of innate and adaptive immunity [16, 17], thus highlighting the possible involvement of cytokines in BA pathogenesis. More specifically, a number of clinical studies uncovered significant increases in systemic levels of several cytokines including inflammatory cytokines (IL-6 and IL-8) [18, 19], chemokines (IP-10 and MCP-1) [20, 21], growth factor (G-CSF) [22] and anti-inflammatory cytokines (IL-1ra and IL-10) [19, 23] in BA patients–especially those with advanced-stage. The above previous findings are in accordance with our findings. In our multivariate logistic regression analyses with adjusting for confounders, BA was shown to be associated with higher plasma levels of 17 out of 27 cytokines including inflammatory cytokines (IL-1β, IL-6, IL-7, IL-8, and IL-9), immunomodulatory cytokines (IL-2 and IL-15), chemokines (eotaxin, IP-10, MCP-1, MIP-1α, and MIP-1β), growth factor (G-CSF), and anti-inflammatory cytokines (IL-1ra, IL-4, IL-5, and IL-10), but significantly correlated with lower plasma IFN-γ levels. In BA patients with unfavorable outcomes, persistent jaundice was found to be significant associated with elevated plasma levels of 6 out 27 cytokines including IL-7, eotaxin, IP-10, IL-1ra, IL-4, and IL-13. Furthermore, significant fibrosis and PH were both significantly associated with higher plasma levels of IL-7, eotaxin, IP-10, and IL-13. Subsequent analysis demonstrated significant associations between those cytokines and clinical parameters including liver stiffness, ALT, AST, TB, DB, ALP, and albumin in BA patients. In contrary to the above findings, reductions in plasma levels of growth factors including PDGF and VEGF were observed to be significantly associated with the presence of BA and jaundice. These findings have been supported by previous clinical studies revealing significant alterations in plasma levels of several growth factors in BA patients compared to healthy controls [10]–particularly a significant decrease in plasma VEGF levels [24]. In hepatitis B virus (HBV)-infected patients, the severity of liver damage and fibrosis was found to be associated with plasma PDGF levels [25], consistent with our additional findings showing associations between plasma levels of VEGF as well as PDGF and clinical parameters, especially liver stiffness in BA patients.

Growth factors, one of the common types of cytokines, are generally known to play regulatory roles in cellular proliferation and differentiation through triggering several biological processes including cell division and survival. In cholestatic hepatopathy, growth factors also induce an imbalance between synthesis and degradation of ECM components via activation of pro- and anti-fibrotic signaling pathways, possibly resulting in liver deterioration and end-stage liver failure [26, 27], thus highlighting the importance of growth factors in BA development. On the basis of the forgoing findings, it has been postulated that cytokines and growth factors could be developed as potential biomarkers of BA. In support of this hypothesis, we conducted the ROC curve analysis unveiling that 4 out of 27 systemic cytokines (IL-8, IP-10, MCP-1, and PDGF) yielded an AUC of more than 0.90, thereby establishing their potential as more sensitive and specific biomarkers for differentiating BA patients from healthy controls than others. In parallel with this finding, the Kaplan-Meier survival analysis showed that high plasma IL-8 levels were significantly associated with reduced survival rate of BA patients following KPE. These results support the conception that plasma IL-8 may have a great potential as a novel biomarker for monitoring BA progression. As IL-8, one of inflammatory cytokines, is recognized as an important mediator of inflammatory and immunological responses via inducing migratory and phagocytic activity in target cells and stimulating angiogenesis, it has been reportedly overexpressed in BA livers, and its mRNA expression was positively associated with hepatic inflammation and liver fibrosis [28], in addition to increased plasma IL-8 levels in BA patients [29]. These previous findings support our additional analysis uncovering that relative *IL-8* mRNA expression was significantly up-regulated in BA livers compared with non-BA livers. Alongside this, up-regulated relative mRNA expressions of *IP-10* and *MCP-1* were found in BA livers. Taken together, our aforementioned findings shed light on not only the significant involvement of cytokines in hepatic and systemic inflammatory responses relevant to BA pathogenesis, but also the potential usefulness of systemic cytokines as novel non-invasive biomarkers of BA, particularly IL-8.

In the light of the above considerations, it is tempting to hypothesize that elevations in systemic levels of inflammatory cytokines and chemokines in BA patients and those with unfavorable outcomes may be indicative of compensatory mechanisms triggered by the body in response to inflammation and immune dysregulation in BA patients. This speculated fate may lead to up-regulated relative mRNA expressions of those cytokines including *IL-8*, *IP-10*, and *MCP-1* in BA livers. For decreases in systemic levels of PDGF and VEGF known as growth factors in BA patients and jaundice patients, the possible reason may be attributed to the disrupted balance between hepatic damage and regenerative capacity upon biliary duct injury, which in turn may decrease systemic and local production of growth factors in BA patients, given an important role of growth factors in regulating cellular proliferation and differentiation during liver regeneration following hepatic injury. These postulated phenomena may help us explain why increased systemic levels of several cytokines and decreased systemic levels of growth factors were both found to be related to clinical parameters and outcomes of BA patients. However, the molecular mechanisms underpinning alterations in systemic levels of cytokines in BA patients remain to be elucidated further.

Despite the significant findings provided herein, we should be mindful of some inherent limitations. The primary disadvantage is that this research was conducted in a cross-sectional manner. For that reason, it is challenging to determine the underlying mechanisms governing the causal connections between elevated plasma levels of cytokines and unfavorable outcomes of post-operative BA patients, in addition to decreased plasma levels of growth factors. To overcome this obstacle, prospective cohort studies are required to verify any associations. Along with this, direct comparisons in plasma cytokine profiles between before and after Kasai operation in BA patients should be carried out. Furthermore, due to the fact that the study participants were from the hospital population rather than the general community, there is a possibility of selection bias if they had any differences in terms of the investigated exposures. Another caveat is the lack of data on co-morbidities of BA, which makes it difficult to interpret our result about whether high plasma IL-8 levels were independently associated with decreased survival of post-operative BA patients. Corresponding to that point, determination on protein localization of candidate cytokines in BA livers and non-BA livers was unachievable, for which immunohistochemical staining needs to be performed. Additionally, owing to ethical concerns, direct comparisons between relative mRNA expressions of candidate cytokines in the liver of healthy controls and those of BA patients were unattainable as a result of being unable to harvest the liver specimens from healthy volunteers.

In spite of its limitations, the strength of this study should be acknowledged. The most notable is the simultaneous measurement of multiple cytokines in a single test sample, which may be more predictive of pathophysiologic, pharmacological, and prognostic importance than assessment of a particular cytokine. As a matter of fact, individual cytokines can play a variety of functions depending on their concentration, availability of receptors, secondary messengers, transcription factors, and the presence of synergistic and/or antagonistic cytokines in the microenvironment. Given that each of these parameters is controlled individually, the concentration of a specific cytokine in a test sample is often inadequate to identify the patient’s health or disease stage. More precisely, the function of any given cytokine is determined by the balance of concurrent synergistic and antagonistic actions of cytokine.

## Conclusions

To the best of our knowledge, this study is the first to demonstrate profiles of 27 systemic cytokines in BA patients and healthy controls. Increased plasma levels of 17 out of 27 cytokines including inflammatory cytokines (IL-1β, IL-6, IL-7, IL-8, and IL-9), immunomodulatory cytokines (IL-2 and IL-15), chemokines (eotaxin, IP-10, MCP-1, MIP-1α, and MIP-1β), growth factor (G-CSF), as well as anti-inflammatory cytokines (IL-1ra, IL-4, IL-5, and IL-10) and decreased plasma levels of IFN-γ and PDGF were found to be significantly associated with the presence of BA. Stratified analyses by disease severity uncovered that elevated plasma levels of IL-7, eotaxin, IP-10, and IL-13 were significantly associated with the presence of unfavorable outcomes including jaundice, fibrosis, and PH in BA patients. Indeed, there were significant associations between systemic levels of several cytokines and clinical parameters including liver stiffness, ALT, AST, TB, DB, ALP, and albumin in BA patients. Based on ROC curve analysis, among 27 cytokines, 4 candidate cytokines including IL-8, IP-10, MCP-1, and PDGF showed a great potential as sensitive and specific biomarkers of BA. Likewise, out of 4 candidate cytokines, high plasma IL-8 levels were detected to be significantly associated with reduced survival of BA patients. Besides increases in systemic levels of 4 candidate cytokines, subsequent analysis demonstrated significantly up-regulated relative mRNA expressions of *IL-8*, *IP-10*, and *MCP-1* in BA livers. Collectively, increased systemic levels of several cytokines including inflammatory cytokines, immunomodulatory cytokines, chemokines, and anti-inflammatory cytokines and decreased systemic levels of growth factors would reflect inflammatory and immune responses related to BA development–especially alterations in plasma levels of IL-8, IP-10, MCP-1, and PDGF. Among 27 cytokines, plasma IL-8 might have great potential as a diagnostic and prognostic biomarker for BA.

## Supporting information

S1 Data (XLSX)

S1 Table Descriptive data of systemic cytokine profiles in healthy controls and BA patients with different subgroups. (DOCX)
