# Supplementary material for: Systemic cytokine profiles in biliary atresia
Source: PLoS One. 2022 Apr 22;17(4):e0267363. doi: 10.1371/journal.pone.0267363 (PMC9032369; doi:10.1371/journal.pone.0267363)
Supplement: S1 Table — (DOCX) [file pone.0267363.s002.docx]

**S1 Table. Descriptive data of systemic cytokine profiles in healthy controls and BA patients with different subgroups.**

|  |  | **Healthy controls**  **(*n*=25)** | **BA patients with different subgroups** | | | | | | |
| --- | --- | --- | --- | --- | --- | --- | --- | --- | --- |
|  |  |  | **Total**  **(*n*=82)** | **Non-jaundice (*n*=51)** | **Jaundice**  **(*n*=31)** | **No fibrosis**  **(*n*=15)** | **Fibrosis**  **(*n*=67)** | **Non-PH**  **(*n*=37)** | **PH**  **(*n*=45)** |
| **Inflammatory cytokines** | | | | | | | | | |
| IL-1β | Mean | 0.12 | 0.45 | 0.25 | 0.61 | 0.17 | 0.45 | 0.36 | 0.42 |
|  | SD | 0.04 | 0.99 | 0.18 | 0.67 | 0.42 | 0.52 | 0.53 | 0.46 |
|  | Median | 0.12 | 0.20 | 0.19 | 0.28 | 0.16 | 0.25 | 0.19 | 0.25 |
|  | IQR (Q1-Q3) | 0.09-0.15 | 0.15-0.30 | 0.13-0.28 | 0.18-0.83 | 0.13-0.19 | 0.17-0.43 | 0.14-0.28 | 0.17-0.36 |
| IL-6 | Mean | 0.64 | 2.49 | 1.44 | 3.05 | 1.07 | 1.07 | 1.40 | 2.57 |
|  | SD | 0.16 | 4.26 | 1.10 | 3.17 | 0.68 | 0.68 | 1.12 | 2.77 |
|  | Median | 0.64 | 1.12 | 1.04 | 1.75 | 0.97 | 0.97 | 1.07 | 1.69 |
|  | IQR (Q1-Q3) | 0.55-0.78 | 0.75-2.23 | 0.80-1.67 | 1.20-3.02 | 0.67-1.12 | 0.98-2.57 | 0.80-1.37 | 1.07-2.66 |
| IL-7 | Mean | 3.48 | 5.23 | 4.29 | 6.90 | 3.61 | 5.86 | 4.97 | 5.70 |
|  | SD | 1.31 | 2.84 | 2.01 | 3.51 | 0.77 | 3.16 | 3.95 | 2.10 |
|  | Median | 3.22 | 4.62 | 3.67 | 6.35 | 3.53 | 6.01 | 3.60 | 6.01 |
|  | IQR (Q1-Q3) | 2.68-4.40 | 3.29-6.82 | 2.99-6.14 | 4.62-8.70 | 3.14-4.18 | 3.45-7.02 | 2.68-5.52 | 3.74-6.82 |
| IL-8 | Mean | 1.60 | 25.37 | 13.52 | 42.62 | 9.45 | 29.34 | 15.55 | 31.99 |
|  | SD | 0.94 | 42.44 | 19.97 | 51.51 | 15.71 | 41.67 | 22.69 | 45.66 |
|  | Median | 1.66 | 7.63 | 5.81 | 16.47 | 2.95 | 9.28 | 5.10 | 10.04 |
|  | IQR (Q1-Q3) | 1.08-1.72 | 3.71-19.62 | 2.53-12.70 | 8.49-71.60 | 1.90-11.07 | 5.99-34.99 | 2.53-14.82 | 6.18-36.68 |
| IL-9 | Mean | 21.23 | 27.91 | 27.73 | 32.16 | 24.99 | 30.80 | 30.35 | 29.11 |
|  | SD | 3.58 | 9.64 | 11.29 | 10.16 | 5.23 | 11.61 | 14.21 | 7.84 |
|  | Median | 22.19 | 26.38 | 26.28 | 28.16 | 25.97 | 27.43 | 26.49 | 27.43 |
|  | IQR (Q1-Q3) | 18.42-24.19 | 21.98-32.00 | 21.61-29.30 | 25.61-34.68 | 21.98-28.68 | 24.87-33.96 | 23.45-29.51 | 24.82-33.65 |
| TNF-α | Mean | 14.05 | 14.76 | 14.49 | 16.51 | 12.64 | 16.05 | 14.90 | 15.67 |
|  | SD | 3.72 | 5.15 | 5.19 | 5.71 | 2.91 | 5.68 | 6.36 | 4.71 |
|  | Median | 13.20 | 14.31 | 13.50 | 16.02 | 12.90 | 15.32 | 13.50 | 15.52 |
|  | IQR (Q1-Q3) | 11.17-16.32 | 10.86-16.52 | 10.35-16.12 | 12.49-17.63 | 9.64-15.32 | 11.27-17.63 | 10.46-16.12 | 12.49-17.33 |
| **Immunomodulatory cytokines** | | | | | | | | | |
| IL-2 | Mean | 0.50 | 1.68 | 1.70 | 2.07 | 1.30 | 2.02 | 1.99 | 1.80 |
|  | SD | 0.44 | 1.31 | 1.18 | 1.88 | 0.36 | 1.63 | 1.97 | 1.08 |
|  | Median | 0.44 | 1.39 | 1.39 | 1.56 | 1.39 | 1.56 | 1.39 | 1.56 |
|  | IQR (Q1-Q3) | 0.16-0.79 | 1.05-1.73 | 1.05-1.86 | 1.22-1.73 | 1.05-1.56 | 1.22-2.32 | 1.05-2.15 | 1.22-1.73 |
| IL-12p70 | Mean | 1.76 | 1.37 | 1.21 | 1.93 | 1.33 | 1.56 | 1.44 | 1.56 |
|  | SD | 0.35 | 1.12 | 0.86 | 1.64 | 0.99 | 1.34 | 1.56 | 1.05 |
|  | Median | 1.71 | 0.93 | 0.93 | 1.42 | 1.03 | 1.13 | 1.03 | 1.13 |
|  | IQR (Q1-Q3) | 1.52-1.91 | 0.83-1.52 | 0.79-1.32 | 0.93-2.45 | 0.93-1.32 | 0.88-1.62 | 0.88-1.52 | 0.93-1.71 |
| IL-15 | Mean | 42.38 | 71.49 | 76.02 | 81.20 | 72.83 | 79.15 | 86.36 | 73.03 |
|  | SD | 23.77 | 31.23 | 31.24 | 39.05 | 33.45 | 34.68 | 45.59 | 22.56 |
|  | Median | 40.82 | 66.75 | 63.38 | 65.61 | 66.67 | 68.92 | 69.45 | 64.55 |
|  | IQR (Q1-Q3) | 25.59-62.80 | 55.27-79.23 | 58.84-86.00 | 57.67-100.56 | 46.39-104.87 | 58.25-86.00 | 61.16-93.83 | 57.67-92.54 |
| IL-17 | Mean | 2.99 | 2.98 | 2.77 | 3.95 | 2.17 | 3.53 | 3.82 | 2.86 |
|  | SD | 0.61 | 2.96 | 1.66 | 5.19 | 0.95 | 3.91 | 5.34 | 1.33 |
|  | Median | 2.98 | 2.55 | 2.23 | 2.77 | 2.07 | 2.77 | 2.66 | 2.77 |
|  | IQR (Q1-Q3) | 2.77-3.19 | 1.91-2.98 | 1.91-3.19 | 2.45-3.19 | 1.91-2.98 | 2.23-3.19 | 2.66-3.30 | 2.12-3.19 |
| IFN-γ | Mean | 23.42 | 6.03 | 9.31 | 7.76 | 6.22 | 9.66 | 9.48 | 8.60 |
|  | SD | 32.98 | 8.11 | 9.43 | 8.22 | 4.03 | 9.84 | 11.95 | 6.55 |
|  | Median | 15.41 | 4.79 | 7.31 | 5.78 | 7.92 | 6.20 | 5.78 | 6.48 |
|  | IQR (Q1-Q3) | 8.93-22.57 | 0.00-7.44 | 4.50-10.27 | 2.41-9.59 | 2.41-10.27 | 4.14-11.46 | 3.84-10.27 | 3.62-11.85 |
| **Chemokines** | | | | | | | | | |
| Eotaxin | Mean | 4.13 | 19.76 | 12.17 | 27.22 | 7.34 | 20.97 | 13.79 | 21.22 |
|  | SD | 4.32 | 15.31 | 10.98 | 13.56 | 7.29 | 14.12 | 14.44 | 13.25 |
|  | Median | 2.89 | 15.56 | 9.35 | 25.84 | 3.85 | 18.84 | 8.92 | 18.84 |
|  | IQR (Q1-Q3) | 1.91-5.66 | 8.00-31.38 | 2.61-20.58 | 16.99-38.51 | 2.71-10.57 | 10.06-31.60 | 2.61-24.01 | 10.93-31.68 |
| IP-10 | Mean | 91.37 | 307.36 | 253.99 | 400.63 | 200.94 | 355.36 | 249.08 | 377.47 |
|  | SD | 50.08 | 205.07 | 147.65 | 276.11 | 108.64 | 239.09 | 132.56 | 262.55 |
|  | Median | 85.03 | 253.22 | 217.86 | 365.37 | 174.96 | 285.96 | 189.08 | 332.67 |
|  | IQR (Q1-Q3) | 54.62-  140.36 | 169.48-406.60 | 160.67-  333.50 | 225.24-465.56 | 103.67-260.30 | 189.08-445.03 | 156.99-346.28 | 200.82-457.42 |
| MCP-1 | Mean | 9.32 | 21.36 | 19.39 | 26.94 | 20.89 | 23.26 | 17.82 | 26.19 |
|  | SD | 4.65 | 13.63 | 9.21 | 21.03 | 11.85 | 16.37 | 9.56 | 17.85 |
|  | Median | 8.98 | 17.93 | 16.68 | 20.39 | 18.55 | 18.80 | 14.79 | 23.36 |
|  | IQR (Q1-Q3) | 6.65-11.97 | 13.71-26.19 | 11.76-25.88 | 16.71-26.00 | 10.98-26.71 | 13.26-25.58 | 11.06-22.35 | 16.65-26.71 |
| MIP-1α | Mean | 0.57 | 2.87 | 3.42 | 2.11 | 1.46 | 3.18 | 3.16 | 2.60 |
|  | SD | 0.15 | 7.91 | 8.74 | 3.81 | 2.28 | 7.77 | 6.21 | 7.62 |
|  | Median | 0.59 | 0.99 | 0.93 | 1.05 | 0.64 | 1.02 | 0.77 | 1.06 |
|  | IQR (Q1-Q3) | 0.44-0.68 | 0.61-1.35 | 0.36-1.31 | 0.77-1.83 | 0.35-1.06 | 0.77-1.38 | 0.36-1.63 | 0.84-1.35 |
| MIP-1β | Mean | 17.36 | 24.97 | 25.03 | 25.10 | 20.37 | 26.18 | 24.05 | 25.65 |
|  | SD | 5.50 | 16.67 | 17.05 | 9.57 | 10.79 | 14.76 | 13.65 | 14.67 |
|  | Median | 16.21 | 20.43 | 18.95 | 23.22 | 17.47 | 22.01 | 18.92 | 21.88 |
|  | IQR (Q1-Q3) | 13.41-18.99 | 15.94-26.30 | 15.93-24.29 | 17.36-29.80 | 14.90-20.82 | 17.30-27.97 | 15.64-26.06 | 17.85-26.44 |
| RANTES | Mean | 14,631.63 | 12,753.37 | 15,590.54 | 17,926.29 | 14,662.79 | 47,908.54 | 15,250.15 | 58,967.52 |
|  | SD | 12,398.26 | 11,513.93 | 14,202.11 | 28,563.72 | 10,221.53 | 193,987.00 | 13,150.18 | 224,706.62 |
|  | Median | 7,571.46 | 8,751.83 | 9,966.13 | 10,395.13 | 11,747.04 | 9,994.67 | 9,526.72 | 9,994.67 |
|  | IQR (Q1-Q3) | 5,548.87-24,793.73 | 3,445.70-18,026.69 | 3,314.01-27,731.51 | 5,065.89-19,241.55 | 5,432.65-24,907.68 | 3,647.32-23,596.41 | 3,514.80-25,901.19 | 4,699.12-20,456.41 |
| **Growth factors** | | | | | | | | | |
| G-CSF | Mean | 24.02 | 67.53 | 72.78 | 66.63 | 37.23 | 82.62 | 88.78 | 62.70 |
|  | SD | 11.92 | 83.29 | 96.78 | 83.10 | 28.68 | 100.51 | 121.86 | 64.78 |
|  | Median | 23.45 | 38.72 | 32.15 | 43.51 | 29.50 | 42.64 | 29.50 | 42.21 |
|  | IQR (Q1-Q3) | 16.15-34.13 | 26.38-60.66 | 26.38-86.81 | 30.16-56.40 | 21.42-35.23 | 29.50-84.30 | 0.72-1.50 | 0.68-1.60 |
| GM-CSF | Mean | 0.57 | 1.27 | 1.09 | 2.03 | 0.79 | 1.66 | 1.52 | 1.45 |
|  | SD | 1.72 | 1.30 | 0.71 | 2.15 | 0.49 | 1.65 | 2.00 | 1.12 |
|  | Median | 0.00 | 0.97 | 0.97 | 1.54 | 0.72 | 1.31 | 0.97 | 1.37 |
|  | IQR (Q1-Q3) | 0.00-0.13 | 0.68-1.44 | 0.61-1.34 | 0.68-2.06 | 0.45-1.04 | 0.79-1.67 | 0.72-1.50 | 0.68-1.60 |
| bFGF | Mean | 6.02 | 6.31 | 6.17 | 6.84 | 4.49 | 7.08 | 6.48 | 6.60 |
|  | SD | 1.63 | 2.91 | 3.14 | 2.95 | 1.64 | 3.19 | 3.45 | 2.92 |
|  | Median | 5.62 | 5.62 | 5.62 | 6.12 | 3.75 | 6.28 | 5.62 | 5.96 |
|  | IQR (Q1-Q3) | 4.92-7.06 | 4.16-7.51 | 3.75-7.21 | 4.74-8.64 | 3.33-5.62 | 4.55-8.92 | 4.16-7.21 | 4.16-8.65 |
| PDGF | Mean | 4,499.85 | 1,619.23 | 1,850.74 | 1,516.10 | 1,672.95 | 1,715.92 | 1,793.76 | 1,647.42 |
|  | SD | 2,059.00 | 1,063.64 | 1,003.25 | 1,024.35 | 903.57 | 1,039.95 | 1,037.67 | 995.48 |
|  | Median | 4,274.19 | 1,298.51 | 1,741.48 | 1,238.57 | 1,303.19 | 1,596.34 | 1,487.38 | 1,507.42 |
|  | IQR (Q1-Q3) | 3,295.93-4,831.56 | 740.86-2,369.51 | 934.25-2,850.08 | 771.83-2,165.51 | 840.43-2,657.19 | 853.42-2,310.39 | 819.52-2,850.08 | 840.43-2,163.19 |
| VEGF | Mean | 97.21 | 95.14 | 107.96 | 76.44 | 75.68 | 107.28 | 121.54 | 86.55 |
|  | SD | 35.50 | 122.50 | 154.14 | 42.30 | 23.47 | 141.33 | 178.59 | 73.55 |
|  | Median | 93.03 | 65.73 | 73.24 | 60.73 | 71.91 | 68.98 | 74.29 | 62.42 |
|  | IQR (Q1-Q3) | 66.27-121.45 | 48.00-85.60 | 56.21-87.59 | 52.44-93.05 | 57.93-90.58 | 52.45-93.05 | 63.46-100.40 | 53.91-92.56 |
| **Anti-inflammatory cytokines** | | | | | | | | | |
| IL-1ra | Mean | 32.38 | 61.80 | 46.83 | 85.34 | 36.40 | 69.39 | 45.89 | 74.22 |
|  | SD | 5.41 | 60.37 | 31.14 | 102.58 | 11.80 | 78.70 | 28.19 | 88.77 |
|  | Median | 32.30 | 52.07 | 39.31 | 62.78 | 37.61 | 59.93 | 41.49 | 59.93 |
|  | IQR (Q1-Q3) | 29.20-35.87 | 35.87-70.59 | 23.26-57.01 | 47.96-75.47 | 28.56-44.80 | 40.71-72.38 | 26.93-58.43 | 42.09-73.71 |
| IL-4 | Mean | 0.14 | 0.43 | 0.31 | 0.63 | 0.18 | 0.51 | 0.46 | 0.43 |
|  | SD | 0.07 | 0.44 | 0.27 | 0.71 | 0.08 | 0.56 | 0.76 | 0.25 |
|  | Median | 0.13 | 0.32 | 0.23 | 0.46 | 0.19 | 0.38 | 0.22 | 0.36 |
|  | IQR (Q1-Q3) | 0.10-0.19 | 0.22-0.50 | 0.14-0.39 | 0.26-0.71 | 0.14-0.24 | 0.23-0.57 | 0.13-0.44 | 0.23-0.49 |
| IL-5 | Mean | 1.24 | 2.78 | 3.07 | 3.49 | 1.91 | 3.61 | 3.55 | 3.07 |
|  | SD | 0.98 | 2.45 | 2.41 | 3.12 | 1.40 | 2.83 | 3.63 | 1.82 |
|  | Median | 1.09 | 2.49 | 2.66 | 2.41 | 1.45 | 2.83 | 2.66 | 2.49 |
|  | IQR (Q1-Q3) | 0.34-2.06 | 1.45-3.34 | 1.45-3.60 | 1.63-4.18 | 0.72-3.17 | 2.06-4.18 | 1.45-3.60 | 1.80-4.18 |
| IL-10 | Mean | 0.54 | 1.99 | 2.34 | 2.58 | 1.43 | 2.74 | 3.05 | 2.07 |
|  | SD | 0.49 | 2.85 | 3.70 | 2.94 | 1.16 | 3.67 | 5.09 | 1.10 |
|  | Median | 0.50 | 1.49 | 1.63 | 1.61 | 1.10 | 1.66 | 1.41 | 1.66 |
|  | IQR (Q1-Q3) | 0.02-0.99 | 1.04-2.00 | 1.10-1.92 | 1.46-2.32 | 0.82-1.72 | 1.43-2.23 | 1.10-2.00 | 1.43-2.23 |
| IL-13 | Mean | 0.54 | 1.32 | 0.78 | 2.53 | 0.30 | 1.79 | 1.18 | 1.70 |
|  | SD | 0.16 | 1.86 | 1.38 | 2.68 | 0.10 | 2.34 | 2.30 | 2.08 |
|  | Median | 0.53 | 0.60 | 0.36 | 1.19 | 0.30 | 0.82 | 0.32 | 0.85 |
|  | IQR (Q1-Q3) | 0.47-0.66 | 0.30-1.29 | 0.21-0.79 | 0.62-3.08 | 0.21-0.30 | 0.47-2.1 | 0.21-0.64 | 0.53-1.95 |

Abbreviations: BA, biliary atresia; bFGF, basic fibroblast growth factor; G-CSF, granulocyte colony stimulating factor; GM-CSF, granulocyte macrophage colony-stimulating factor; IFN, interferon; IL, interleukin; IL-1ra, anti-inflammatory cytokines including IL-1 receptor antagonist; IP, IFN-γ-induced protein; MCP, monocyte chemoattractant protein; MIP, macrophage inflammatory protein; PDGF, platelet-derived growth factor; PH, portal hypertension; RANTES, Regulated on Activation, Normal T Expressed and Secreted; TNF, tumor necrosis factor; VEGF, vascular endothelial growth factor.
